# Tween-20 transiently changes the surface morphology of PK-15 cells and improves PCV2 infection

**DOI:** 10.1186/s12917-018-1457-5

**Published:** 2018-04-24

**Authors:** Tao Hua, Xuehua Zhang, Bo Tang, Chen Chang, Guoyang Liu, Lei Feng, Yang Yu, Daohua Zhang, Jibo Hou

**Affiliations:** 1 Institute of Veterinary Immunology & Engineering, Jiangsu Academy of Agricultural Sciences, Nanjing, 210014 China; 2 National Research Center of Engineering and Technology for Veterinary Biologicals, Jiangsu Academy of Agricultural Science, Nanjing, 210014 China; 3 Key lab of Food Quality and Safety of Jiangsu Province—State Key laboratory Breeding Base, Jiangsu Academy of Agricultural Science, Nanjing, 210014 China; 4 Jiangsu Co-innovation Center for Prevention and Control of Important Animal Infectious Diseases and Zoonoses, Yangzhou, 225009 China

**Keywords:** Nonionic surfactant, Tween-20, PCV2, Viral infection, Cellular morphologic change

## Abstract

**Background:**

Low concentrations of nonionic surfactants can change the physical properties of cell membranes, and thus and in turn increase drug permeability. Porcine circovirus 2 (PCV2) is an extremely slow-growing virus, and PCV2 infection of PK-15 cells yields very low viral titers. The present study investigates the effect of various nonionic surfactants, namely, Tween-20, Tween-28, Tween-40, Tween-80, Brij-30, Brij-35, NP-40, and Triton X-100 on PCV2 infection and yield in PK-15 cells.

**Result:**

Significantly increased PCV2 infection was observed in cells treated with Tween-20 compared to those treated with Tween-28, Tween-40, Brij-30, Brij-35, NP-40, and Triton X-100 *(p* < 0.01). Furthermore, 24 h incubation with 0.03% Tween-20 has shown to induce significant cellular morphologic changes (cell membrane underwent slight intumescence and bulged into a balloon, and the number of microvilli decreased), as well as to increase caspase-3 activity and to decrease cell viability in PCV2-infected PK-15 cells cmpared to control group; all these changes were restored to normal after Tween-20 has been washed out from the plate.

**Conclusion:**

Our data demonstrate that Tween-20 transiently changes the surface morphology of PK-15 cells and improves PCV2 infection. The findings of the present study may be utilized in the development of a PCV2 vaccine.

## Background

Porcine circovirus 2 (PCV2), which belongs to family Circoviridae, genus Circovirus, is the smallest non-enveloped, single-stranded, circular DNA virus that replicates autonomously. PCV2 was identified in the mid-1990s as the causative agent of post-weaning multisystemic wasting syndrome (PMWS), and is one of the most economically important viral pathogens among all major swine-producing countries [1]. Previous studies have shown that viral antigens, RNA transcripts, and progeny viruses all increase in a time-dependent manner during productive infection [1, 2]. The PK-15 cell line, which is widely used in PCV2 propagation, does not undergo efficient viral infection [3]. In China, several virus-inactivated vaccines derived from Chinese PCV2 strains have been extensively utilized in controlling PMWS and other porcine circovirus-associated disease [4]. Therefore, increasing the infection and replication of PCV2 in PK-15 cells may potentially facilitate in vaccine production, particularly in terms of efficiency and profitability. Several methods of increasing viral yield have been reported [5–11].

Previous studies have suggested that nonionic surfactants increase drug permeability through the cell membranes, thereby improving bioavailability [12–16]. When present at low concentrations, these surfactants are incorporated into the lipid bilayer, forming polar defects that alter the physical properties of cell membranes. In addition, nonionic surfactants promote membrane transport of various materials such as hydrocortisone and lidocaine across hairless mouse skin as mediated by Tween-80 [15], 5-flourouracil across hairless mouse skin by 6-fold using Tween-20 [16], and fluorescein in corneal tissues by Tween-20 and Brij-35 [13]. The aim of the present study was to investigate the effect of Tween-20, Tween-28, Tween-40, Tween-80, Brij-30, Brij-35, NP-40, and Triton X-100 on PCV2 infection and yield in PK-15 cells.

## Methods

### Virus, cells, and reagents

PCV2 strain DBN-SX07 was isolated from a piglet (Piglet was bought from a commercial pig farm in China’s Shanxi province and was euthanized by an anesthetic overdose with the pentobarbital before collected the samples) in China (GenBank Accession No. FJ660968). PCV-free PK-15 cells, purchased from the China Institute of Veterinary Drug Control (Beijing, China), were maintained in minimum essential medium (MEM) (Gibco, Carlsbad, CA, USA) supplemented with 5% calf serum (CS) (Gibco Carlsbad, CA, USA), 100 U/mL penicillin (Sigma-Aldrich, St. Louis, MO, USA) and 0.1 mg/mL streptomycin (Sigma-Aldrich, St. Louis, MO, USA). Nonionic surfactants Tween-20, Tween-28, Brij-30, Brij-35 NP-40, and Triton X-100 were obtained from Sigma (St. Louis, MO, USA), while Tween-40 and Tween-80 were purchased from CRODA (Shanghai, China).

### Effect of different nonionic surfactants on PCV2 infection in PK-15 cells

The highest concentrations of each nonionic surfactant that does not affect PK-15 cell viability 24 h after incubation period were used (Table1). PK-15 cells were seeded into the wells of a 96-well plate (Corning Incorporated, Shanghai, China) at a density of 2 × 10^5^ cells/mL, with a volume of 100 μL for each well. After 24 h, Cell culture medium was then removed, and cells were consequently incubated for 23 h in a 5% CO2 incubator at 37 °C with or without different concentrations of nonionic surfactants (diluted in cell culture medium without CS) (Table 1), following the incubation with PCV2 at a multiplicity of infection (MOI) of 0.5 for 1 h at 37 °C and 5% CO_2_ in the presence or absence of nonionic surfactants. 24 h post treatment, the viral inoculum and nonionic surfactants were washed off and PK-15 cells were further incubated in cell culture medium containing 2% CS, 100 U/mL penicillin and 0.1 mg/mL streptomycin at 37 °C with 5% CO_2_. 72 h later, the untreated and treated cells were fixed in cold 80% acetone (Nanjing Chemical Reagent CO., Nanjing, China) at 4 °C for 10 min. PCV2-infected PK-15 cells were identified using an indirect immunofluorescence assay (IFA) as described Section IFA. PCV2-infected PK-15 cells were counted and analyzed using fluorescence microscope Zeiss Axio Vert (Carl Zeiss AG, Oberkochen, Germany). The number of infected cells among the untreated cells was used as a reference, and all results were expressed as relative percentages to this reference. Data were expressed as the means of at least three independent experiments.

**Table 1:** Effect of nonionic surfactants on PCV2 infection in PK-15 cells

Agent	Concentration	Relative % of PCV2-infected cells (± S.D.)^**α**^
Tween-20	0.03%	880 ± 128
	0.02%	715 ± 152
	0.01%	380 ± 128
Tween-28	0.1%	140 ± 18
	0.05%	110 ± 20
	0.03%	90 ± 10
Tween-40	0.1%	175 ± 52
	0.05%	180 ± 37
	0.03%	145 ± 15
Tween-80	0.2%	430 ± 75
	0.1%	350 ± 60
	0.03%	270 ± 45
Brij-30	0.0005%	175 ± 35
	0.0003%	190 ± 43
	0.0001%	230 ± 45
Brij-35	0.0005%	250 ± 30
	0.0003%	458 ± 84
	0.0001%	469 ± 60
NP-40	0.02%	150 ± 45
	0.01%	400 ± 75
	0.005%	220 ± 58
Triton X-100	0.02%	232 ± 58
	0.01%	400 ± 13
	0.005%	460 ± 67

^α^The percentages of PCV2-infected PK-15 cells following treatment with different agents are expressed relative to the number of PCV2-infected cells in untreated PK-15 cells. The data are expressed as the mean ± standard deviation of three experiments

### Immunofluorescence assay (IFA) analysis

PK-15 cells, which were inoculated with PCV2 in 96-well culture plates, were rinsed with phosphate buffered saline (PBS) (Wuhan Goodbio technology CO., Nanjing, China) nd fixed with cold 80% acetone for 10 min at 4 °C. The cells were washed, and then incubated for 1 h with anti-PCV2 antibody (VMRD, USA) diluted 1:200 in PBS with 0.05% Tween 20 (PBS-T) at 37 °C. After washing with PBS-T, cells were incubated with Staphylococcal protein A conjugated with fluorescein (1:50 diluted in PBS-T) as a secondary antibody (Boshide, Wuhan, China) for 45 min at 37 °C. After five rinses, cells were observed under a fluorescence microscope Zeiss Axio Vert (Carl Zeiss AG, Oberkochen, Germany).

### Kinetics of PCV2 replication in PK-15 cells treated with nonionic surfactants

PK-15 cells were seeded into the wells of a 24-well plate (Corning Incorporated, Shanghai, China) at a density of 2 × 10^5^ cells/mL, with a volume of 0.5 mL for each well. After 24 h, the culture medium was removed, and cells were washed and incubated for 23 h in a 5% CO2 incubator at 37 °C with or without different concentrations of nonionic surfactants diluted in cell culture medium without CS (Table 2). Subsequently, PK-15 cells were inoculated with PCV2 (MOI = 0.5) for 1 h at 37 °C and 5% CO_2_ in the presence or absence of nonionic surfactants. After 24 h of treatment, the viral inoculum and nonionic surfactants were washed off and PK-15 cells were further incubated in cell culture medium containing 2% CS, 100 U/mL penicillin and 0.1 mg/mL at 37 °C with 5% CO_2_. The medium and cells from triplicate wells of each inoculation group were harvested every 24 h through 96 h post treatment (hpt) and stored at − 70 °C until virus titration.

**Table 2:** Kinetics of PCV2 replication in PK-15 cells treated with or without different nonionic surfactants

Agent	Concentration	PCV2 titer log_10_ (TCID_50_/mL)			
		24 hpt^a^	48 hpt	72 hpt	96 hpt
Control	0	1.3	2.7	3.2	3.3
Tween-20	0.03%	2.7	4.0	4.5	4.5
Tween-28	0.1%	1.5	2.7	3.2	3.3
Tween-40	0.1%	1.7	3	3.3	3.5
Tween-80	0.2%	2.2	3.5	3.8	3.7
Brij-30	0.0003%	1.7	2.8	3.5	3.5
Brij-35	0.0003%	2.3	3.3	3.8	3.8
NP-40	0.01%	2.2	3.3	3.5	3.5
Triton X-100	0.01%	2.2	3.3	3.8	3.7

^a^hours post-treatment

### Scanning Electron microscopy (SEM) analysis

PK-15 cells were grown on 18 × 18 mm coverslips (Sail Brand, Guangdong, China) in 6-well plates (Corning Incorporated, Shanghai, China) at a density of 2 × 10^5^ cells/mL, with a volume of 2 mL for each well. After 24 h, the culture medium was removed. PK-15 cells were then washed and incubated for 23 h in a 5% CO_2_ incubator at 37 °C with or without 0.03% Tween-20 diluted in cell culture medium without CS. After that, PK-15 cells were inoculated with PCV2 (MOI = 0.5) for 1 h at 37 °C and 5% CO_2_ in the presence or absence of 0.03% Tween-20. 24 h post treatment, the viral inoculum and nonionic surfactants were washed off and PK-15 cells were further incubated in cell culture medium containing 2% CS, 100 U/mL penicillin and 0.1 mg/mL streptomycin at 37 °C with 5% CO_2_. After 0, 24, 48, and 72 h post Tween-20 treatment, cells were prepared for SEM analysis. Briefly, the PK-15 cells were washed with PBS, followed by fixation in 3% glutaraldehyde (Sigma-Aldrich, St. Louis, MO, USA) for 2 h at room temperature. After three washes with distilled water for 30 min at room temperature, the cells were serially dehydrated in ethanol (Nanjing Chemical Reagent Co., Nanjing, China) (30%, 70%, 96%, 3 × 100%, 15 min for each step), critical point-dried, sputter-coated with gold particles, and stored in a desiccator until observation. Finally, all specimens were examined in a Zeiss EVO-LS10 SEM (Carl Zeiss AG, Oberkochen, Germany).

### Measurement of caspase-3 activity

PK-15 cells were placed in the wells of a 6-well plate at a density of 2 × 10^5^ cells/mL, with a volume of 2 mL for each well. After 24 h, the culture medium was removed. PK-15 cells were washed and incubated for 23 h in a 5% CO2 incubator at 37 °C with or without 0.03% Tween-20 diluted in cell culture medium without CS. Afterwards, PK-15 cells were inoculated with PCV2 (MOI = 0.5) for 1 h at 37 °C and 5% CO_2_ in the presence or absence of 0.03% Tween-20. After 24 h of treatment, the viral inoculum and nonionic surfactants were washed off and PK-15 cells were further incubated in cell culture medium containing 2% CS, 100 U/mL penicillin and 0.1 mg/mL streptomycin at 37 °C with 5% CO_2_. The PCV2-infected PK-15 cells were collected at 0, 24, 48, and 72 h post Tween-20 treatment. Caspase-3 activity was determined by a colorimetric assay, which was based on the ability of caspase-3 to convert acetyl-Asp-Glu-Val-Asp p-nitroanilide (Ac-DEVD-pNA) into a yellow formazan product (p-nitroanilide). An increase in the absorbance at a wavelength of 405 nm was indicative of caspase-3 activation. The culture medium and PK-15 cells were collected at indicated times. The cells were rinsed with cold PBS, and lysed with lysis buffer (100 μL/2 × 10^6^ cells) for 15 min on ice. The cell lysates were centrifuged at 18,000 *g* for 10 min at 4 °C. Caspase-3 activity was determined using a caspase-3 activity kit (Beyotime Institute of Biotechnology, Nantong, China) following the manufacturer′s protocol.

### Cell viability measurement

The effect of Tween-20 on cell viability was determined by using the MTT [3-(4, 5-dimethylthiazol-2-yl)-2,5-diphenyl tetrazolium bromide, MTT] assay following the manufacturer’s instructions (Merk Millipore, Shanghai, China). PK-15 cells were seeded into a 96-well plate at a density of 2 × 10^5^ cells/mL, with a volume of 100 μL for each well.. After 24 h, PK-15 cells were washed and incubated for 23 h in a 5% CO_2_ incubator at 37 °C with or without 0.03% Tween-20 diluted in cell culture medium without CS. Afterwards, PK-15 cells were inoculated with PCV2 (MOI = 0.5) for 1 h at 37 °C and 5% CO_2_ in the presence or absence of 0.03% Tween-20. 24 h later, the viral inoculum and nonionic surfactants were washed off and PK-15 cells were further incubated in cell culture medium containing 2% CS, 100 U/mL penicillin and 0.1 mg/mL streptomycin at 37 °C with 5% CO_2_. The PCV2-infected PK-15 cells were collected at 0, 24, 48, and 72 h post treatment with Tween-20. Approximately 10 μL of MTT (5 mg/mL) was added onto each well of the 96-well plate and then incubated for another 4 h at 37 °C. After incubation, the culture medium was removed, and 100 μL of acidified isopropanol (Sigma-Aldrich, St. Louis, MO, USA) was added to each well to dissolve the precipitate at room temperature. Absorbance was measured at a wavelength of 570 nm using a Stat Fax-2100 spectrophotometer (Awareness Technology, Inc., USA). Each treatment was performed in triplicate, and the viability of treated cells was expressed as the relative percentage of live cells relative to that of the untreated control cells.

### Statistical analysis

Statistical analysis was performed using GraphPad PRISM software (version 5.02 for Windows; GraphPad Software, Inc.). The data were analyzed to establish their significance using one-way or two-way ANOVA followed by a least-significant difference test. The data were expressed as the mean ± SD. Differences were regarded as significant at *p* < 0.01.

## Results

### Effect of different nonionic surfactant on PCV2 infection

**Fig. 1:**
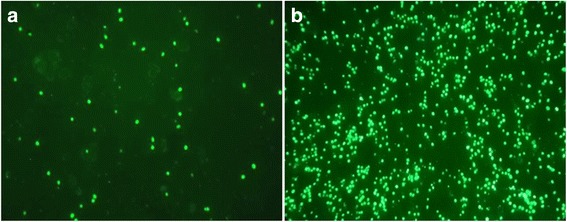
Effect of Tween-20 on PCV2 infection in PK-15 cells. PK-15 cells were treated with or without 0.03% Tween-20 for 24 h, and simultaneously infected PCV2 (MOI = 0.5) for 1 h. After a 24-h treatment, the mixed solution of Tween-20 and PCV2 was washed off and the PK-15 cells were further incubated in cell culture medium containing 2% CS. After 72 h post treatment, the PCV2-infected cells were assessed using an immunofluorescence assay. The number of PCV2-infected cells from PK-15 cells treated with 0.03% Tween-20 was significantly higher compared to PCV2-infected PK-15 cells without Tween-20 treatment (*p* < 0.01, Table 1). **a** PCV2-infected PK-15 cells without Tween-20 treatment as control. **b** PCV2-infected PK-15 cells treated with Tween-20. Magnification: × 100

The PK-15 cells were treated with different concentrations of nonionic surfactants to investigate its effect on PCV2 infection (Table 1). The relative number of PCV2-infected cells in PK-15 cells were 880 ± 128%, 140 ± 18%, 180 ± 37%, 430 ± 75%, 230 ± 45%, 469 ± 60%, 400 ± 75%, and 460 ± 67% when PK-15 cells were treated with 0.03% Tween-20, 0.1% Tween-28, 0.05% Tween-40, 0.2% Tween-80, 0.0001% Brij-30, 0.0001% Brij-35, 0.01% NP-40, and 0.005% Triton X-100, respectively (Table 1). 0.03% Tween-20 treatment increased PCV2-infected PK-15 cells by up to 8.8 times compared to untreated PK-15 cells. The number of PCV2-infected cells from PK-15 cells treated with 0.03% Tween-20 was significantly higher compared to those treated with Tween-28, Tween-40, Brij-30, Brij-35, NP-40, Triton X-100, and untreated PK-15 cells (*p* < 0.01, Table 1 and Fig. 1). The number of PCV2-infected cells in PK-15 cells treated with Tween-80, Brij-35, NP-40 and Triton X-100 was significantly higher than the untreated PK-15 cells, but significantly lower than PK-15 cells treated with Tween-20 (Table 1). Furthermore, no significant changes were observed when treating cells with Tween-28 or Tween-40 compared to untreated PK-15 cells (Table 1). The relative number of PCV2-infected cells in PK-15 cells were 880 ± 128%, 715 ± 152% and 380 ± 128% when PK-15 cells were treated with 0.03%, 0.02% and 0.01% Tween-20, respectively (Table 1). After increasing the concentration of Tween-20 (> 0.03%) for 24 h, cell viability was significantly affected and the number of PCV2-infected cells decreased (data not shown). When nonionic surfactants exceeded the highest concentration in Table 1, cell viability would be significantly affected and the number of PCV2-infected cells decreased (data not shown). The highest concentrantion of Brij-35, NP-40 and Triton X-100 in Table 1 didn’t show the highest effect on promoting the number of PCV2-infected cells. Some function of cells may be affected at the highest concentration of Brij-35, NP-40 and Triton X-100 in Table 1.

### Kinetics of PCV2 replication in PK-15 cells

The kinetics of PCV2 replication was determined in PK-15 cells treated with or without different nonionic surfactants (Table 2). After the initial infection, the replication levels of PCV2 were detected in PK-15 cells. The results showed that all viral stocks, originating from the infected cells, had low initial titers (Table 2). 72 h post-treatment, the PCV2 titers of the PK-15 cells treated with 0.03% Tween-20 rapidly increased and were higher (10^4.5^ TCID_50_/mL) compared to other treatments (Table 2).

### Assessment of morphologic changes in PK-15 cells

**Fig. 2:**
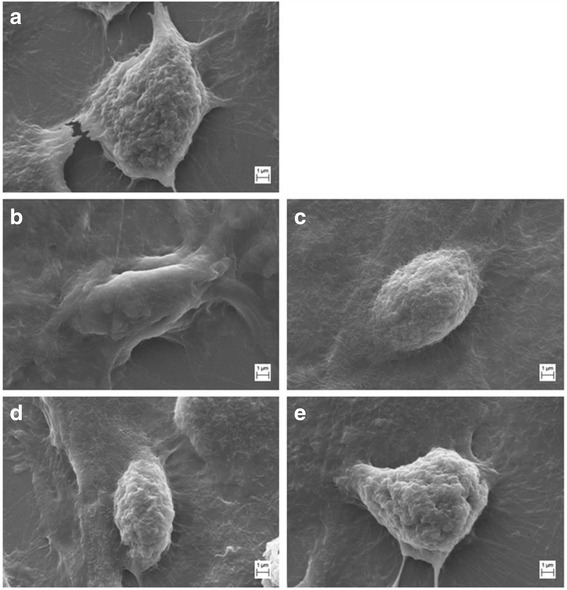
The morphologic changes of PCV2-infected PK-15 cells were observed by SEM at the indicated times of 0.03% Tween-20 post treatment. **a** PCV2-infected PK-15 cells without Tween-20 treatment as control. **b** PCV2-infected PK-15 cells at 0 h post Tween-20 treatment. **c** PCV2-infected PK-15 cells at 24 h post Tween-20 treatment. **d** PCV2-infected PK-15 cells at 48 h post Tween-20 treatment. **e** PCV2-infected PK-15 cells at 72 h post Tween-20 treatment. Bar, 1 μm

PK-15 cellular morphologic changes at 0, 24, 48, and 72 h post 0.03% Tween-20 treatment were analyzed using SEM (Fig. 2). The surface of PCV2-infected PK-15 cells without Tween-20 treatment showed an abundance of microvilli and was rough in appearance. After 24 h treatment with Tween-20, the cells membrane of the PK-15 cells exhibited slight intumescence and bulged into a balloon, and the number of microvilli significantly decreased; while, all those changes were restored to normal after Tween-20 has been washed out from the plate. These findings indicated that the surface structure of PK-15 cells recovered after transient treatment with 0.03% Tween-20.

### Tween-20 transiently promotes caspase-3 activation

**Fig. 3:**
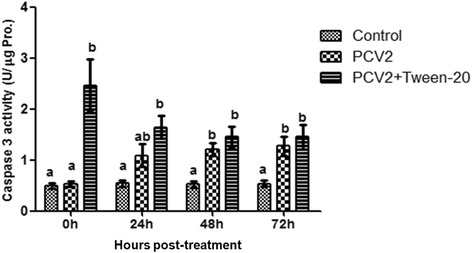
Effect of 0.03% Tween-20 on caspase-3 activation of PCV2-infected PK-15 cells. Cell lysates were harvested at the indicated post treatment times. Caspase-3 activity was measured by using a colorimetric assay based on the ability of caspase-3 to convert Ac-DEVD-pNA into a yellow formazan product. The data are expressed as the mean ± SD (*n* = 3). Within each time point, means with different letters (a, b, c) are significantly different from each other (*p* < 0.01)

PCV2 has been shown to induce apoptosis in cultured cells through activation of caspase-8, followed by activation of the caspase-3 pathway [17]. Tween-20 can induce membrane damage and initiate apoptosis [18]. To determine whether Tween-20 improves PCV2-induced apoptosis, cell lysates were harvested at various time points and assayed for caspase-3 activity. Following infection with PCV2 alone, a time-dependent increase in the cleavage of ρNA (a product of caspase-3 cleaving Ac-DEVD-ρNA) was observed throughout the course of post-infection. Caspase-3 activity in PK-15 cells were 0.53 ± 0.05, 1.09 ± 0.22, 1.21 ± 0.12, and 1.27 ± 0.19 U/mg protein (Pro.) when cells were infected with PCV2 alone at 0, 24, 48, and 72 h post PCV2 infection, respectively (Fig. 3). The percentage of increase caspase-3 activity were 110 ± 15%, 200 ± 13%, 234 ± 32%, and 239 ± 22% when PK-15 cells were infected with PCV2 alone compared to the control cells at 0, 24, 48, and 72 h post PCV2 infection, respectively. This indicated that caspase-3 was progressively activated by PCV2 infection (Fig. 3). Consequently, we examined the effect of 0.03% Tween-20 on caspase-3 activity in PCV2-infected cells. Caspase-3 activity in PK-15 cells were 2.46 ± 0.51, 1.64 ± 0.22, 1.45 ± 0.19, and 1.45 ± 0.24 U/mg Pro. when PK-15 cells were treated 0.03% Tween-20 and simultaneously infected PCV2 at 0, 24, 48, and 72 h post post-treatment, respectively (Fig. 3). The percentage of increase caspase-3 activity was 503 ± 26%, 304 ± 22%, 279 ± 22%, and 273 ± 0.24% when PK-15 cells were treated 0.03% Tween-20 and simultaneously infected PCV2 compared to the control cells at 0, 24, 48, and 72 h post-treatment, respectively. The percentage of increase caspase-3 activity in PK-15 cells were 462 ± 84%, 152 ± 19%, 119 ± 7%, and 114 ± 1% when PK-15 cells were treated 0.03% Tween-20 and simultaneously infected PCV2 compared to untreated PCV2-infected cells at 0, 24, 48, and 72 h post-treatment, respectively. Caspase-3 activity in PCV2-infected cells treated 0.03% Tween-20 significantly increased compared to that in the PCV2-infected cells at 0 h post-treatment, while its activity returned to normal after removing Tween-20 from the plate (no significant difference in caspase-3 activity was observed between treated and untreated PCV2-infected cells at 24, 48, and 72 h post Tween-20 treatment). To sum up, these findings were indicative of a decrease in caspase-3 activation after removal of Tween-20.

### Transient treatment with 0.03% Tween-20 does not significantly affect cell viability

**Fig. 4:**
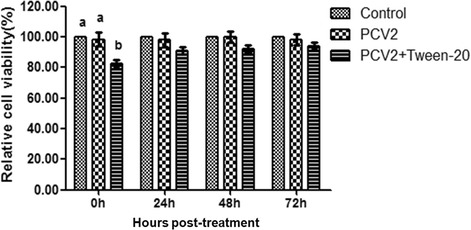
Effect of 0.03% Tween-20 on the viability of PCV2-infected PK-15 cells. Cell viability was determined by using an MTT assay at the indicated post treatment times. The percentage of relative cell viability is expressed as themean ± SD (n = 3). Within each time point, values with different letters (a, b) are significantly different from each other (*p* < 0.01)

MTT assay was used to examined whether 0.03% Tween-20 affects cell viability (Fig. 4). The PK-15 cells infected with PCV2 did not show an adverse change in cell viability compared to that in the control cells. Importantly, 0 h post- treatment with Tween-20 significantly decreased cell viability, which was then restored to normal after removing Tween-20 from the plate (no significant difference was observed between treated anduntreated PCV2-infected cells (*p* > 0.01)). These findings indicate that cell viability was increased when Tween-20 was washed off.

## Discussion

Nonionic surfactants are a category of surfactants with uncharged hydrophilic and hydrophobic heads [19]. Nonionic surfactants can form structures in which hydrophilic heads are oriented opposite to the aqueous solutions, and hydrophobic heads opposite to the organic solutions. Based on this property, low concentrations of these surfactants can be incorporated into the lipid bilayer, forming polar defects that alter the physical properties of the cell membranes. When the lipid bilayer is saturated, mixed micelles begin to form, resulting in the removal of phospholipids from the cell membranes and membrane solubilization. Marsh and Maurice [13] have evaluated the effect of nonionic surfactants on corneal permeability and toxicity in humans, and found that Tween-20 and Brij-35 are the most effective in increasing corneal permeability. In the present study, the effects of nonionic surfactants Tween-20, Tween-28, Tween-40, Tween-80, Brij-30, Brij-35, NP-40, and Triton X-100 on PCV2 infection in PK-15 cells were investigated. Interestingly, Tween-20 treatment significantly increased the number of PCV2-infected cells compared to control and other nonionic surfactant treatments groups (*p* < 0.01, Table 1, and Fig. 1).

Cytotoxicity is an inherent property of various nonionic surfactants [12, 18, 20–22]. These nonionic surfactants can induce membrane damage and initiate apoptosis. However, a previous study has shown that their cytotoxicity could be reduced by using the appropriate type and/or number of side chains [20]. Tween-80 has the lowest cytotoxicity in normal human fibroblast cultures compared to Texapon N40, Tween-60, Texapon K1298, Triton X-100, and benzethonium chloride [22]. The cytotoxicity of nonionic surfactants can be further reduced using lower concentrations [18]. The application of Tween-20 concentration range of 0.013%–0.025% has shown to exert considerable cytotoxicity in both multidrug resistance cell lines and their parental cells after 48 h exposure. Tween-20 at concentrations < 0.01% is non-toxic to all cells, showing > 90% cell survival. In the present study, 24 h treatment with 0.03% Tween-20 induced cellular morphologic changes (cell membrane underwent slight intumescence and bulged into a balloon, and the number of microvilli decreased) (Fig. 2), increased caspase-3 activity (Fig. 3) and decreased cell viability (Fig. 4) in PCV2-infected PK-15 cells compared to control group; while all these changes were restored to normal after Tween-20 has been washed out from the plate.

Due to the low replication efficiency of PCV2, researchers have adopted various ways of improving virus titers [5–11]. PCV multiplication is inducible by treating infected cell cultures with D-glucosamine (D-G) [6]. Cholesterol removal enhances PCV2 replication in epithelial cells treated with methyl-β-cyclodextrin [9]. Some studies have shown that the number of PCV2-infected cells increases after treating PK-15 cells with either interferon-gamma, or inhibitors of endosomal-lysosomal system acidification such as ammonium chloride, chloroquine diphosphate, and monensin [5, 8]. The present study showed that PK-15 cells treated with Tween-20 significantly increased PCV2 infection compared to other nonionic surfactants, including Tween-28, Tween-40, Tween-80, Brij-30, Brij-35, NP-40, and Triton X-100 and untreated PK-15 cells (*p* < 0.01).

## Conclusions

The present study examined the effects of nonionic surfactants on PCV2 infection. We demonstrated that PCV2-infected PK-15 cells treated with Tween-20 showed an increase in PCV2 infection and yield compared to other nonionic surfactants such as Tween-28, Tween-40, Tween-80, Brij-30, Brij-35, NP-40, and Triton X-100 and untreated PK-15 cells. Furthermore, SEM analysis showed that Tween-20 could transiently change the surface morphology and structure of PK-15 cells to improve PCV2 infection. After transient treatment with Tween-20, SEM and caspase-3, and MTT assays indicated a restoration of the surface structure and viability of PK-15 cells. Therefore, PK-15 cells treated with Tween-20 may be potentially used in increasing PCV2 infection, which in turn may facilitate the vaccine production.

## Abbreviations

Ac-DEVD-pNA: acetyl-Asp-Glu-Val-Asp p-nitroanilide
CS: Calf serum
hpt: Hours post treatment
IFA: Immunofluorescence assay
MEM: Minimum essential medium
MOI: multiplicity of infection
PBS: Phosphate buffered saline
PBS-T: PBS with 0.05% Tween 20
PCV2: Porcine circovirus 2
Pro: Protein.
SEM: Scanning electron microscopy
